# Shape and Variability of the Normal Medial Coronoid Process by Computed Tomography in Young Adult Labrador Retrievers

**DOI:** 10.1111/vru.70092

**Published:** 2025-09-25

**Authors:** Luzanne van der Laan, Robert M. Kirberger, Geoffrey T. Fosgate, Christelle Le Roux

**Affiliations:** ^1^ Department of Companion Animal Clinical Studies Faculty of Veterinary Science University of Pretoria Onderstepoort South Africa; ^2^ Department of Production Animal Studies Faculty of Veterinary Science University of Pretoria Onderstepoort South Africa; ^3^ Department of Veterinary Clinical Sciences Long Island University Brookville New York USA

**Keywords:** canine | elbow dysplasia | Hounsfield units | medial compartment disease | radial head density

## Abstract

Medial coronoid process disease (MCPD) is the most frequently observed cause of elbow dysplasia, resulting in lameness in young, fast‐growing large‐breed dogs, including Labrador Retrievers (LRs). Computed tomography (CT) is the diagnostic imaging modality of choice for evaluating the medial coronoid process (MCP), as it is noninvasive and eliminates superimposition of the process by the radial head. This retrospective descriptive study aimed to describe the shape of the normal MCP on CT, to assess its variability within the LR breed, and to determine the normal Hounsfield units (HUs) of the MCP, medial radial head (MRH), and lateral radial head (LRH). Normal elbow CT studies of 51 South African guide dog LRs were reviewed. Using a repeatable imaging alignment technique, three principal MCP shapes were identified: ovoid, triangular, and softly pointed and were found to be dependent on the level of assessment. Males had significantly lower mean MCP HU compared to females. The mean HU of the MRH was consistently higher than the LRH and was also greater in attenuation on subjective assessment. Measuring MCP and radial head HU too proximally was suboptimal, as volume averaging was frequently encountered. The results of this study showed that although different alignment techniques may result in HU variations, they will not affect the HU to such an extent that the MCP would be misclassified as abnormal.

**Abbreviations**
ED: elbow dysplasia
GR: Golden Retriever
LR: Labrador Retriever
LRH: lateral radial head
MCP: medial coronoid process
MCPD: medial coronoid process disease
MRH: medial radial head

## 1 Introduction

Elbow dysplasia (ED) is a debilitating hereditary condition of young large and giant‐breed dogs, such as the Labrador Retriever (LR) [1, 2, 3, 4]. Four primary lesions can occur on their own or concurrently, namely, medial coronoid process disease (MCPD), ununited anconeal process (UAP), joint incongruity, and osteochondritis dissecans (OCD) of the humeral trochlea [3, 4, 5, 6].

The aetiopathogenesis of MCPD is not yet fully understood, despite being the most frequent cause of ED [1, 7, 8, 9]. Joint incongruity and an increase in trans‐articular pressure have been proposed as the underlying cause for the development and progression of MCPD [7, 8, 9]. However, MCPD is not always associated with apparent joint incongruity, with continued growth after MCPD development proposed as the reason for spontaneous resolution of joint incongruity [4, 10, 11]. Other studies reported no link between MCPD and joint incongruity or noted that incongruity was not always present with a fragmented medial coronoid process (MCP) [12, 13]. MCPD might, therefore, develop as a primary lesion rather than purely as a result of incongruity, which may develop later and is therefore unlikely to be the sole cause for the development of MCPD [13, 14].

MCPD is characterized by chondromalacia, fragmentation, fissures, abnormal shape, sclerosis, associated radial incisure irregularities, and radial head sclerosis [15, 16, 17, 18, 19]. The genetic complexity of ED and the lack of available genetic testing mean veterinarians must rely heavily on imaging findings, and due to its heritability, it is important to identify and remove affected animals from the breeding stock [20, 21, 22, 23].

Computed tomography (CT) is the diagnostic imaging modality of choice for evaluating the MCP, as it is noninvasive and eliminates superimposition of the process by the radial head. The ability to adjust window width (WW) and level (WL) is useful for the assessment of this complex joint [21, 24, 25]. Groth et al. have proposed that normal variation in MCP shape on CT can be expected, reflecting differences between breeds and individuals from the same breed [26]. These authors did not elaborate further but reported that it was difficult to distinguish normal variation from disease based on MCP shape alone.

Only one article has endeavored to describe the normal MCP shape [27]. This study evaluated three breeds, including LR, Golden Retriever (GR), and German Shepherd dogs, which were included on the basis of a radiographic ED grading of zero. Four distinct MCP shapes were identified: rounded, pointed, flattened, and irregular, with MCP size being sex‐dependent. The MCP Hounsfield units (HUs) were found to be significantly sex‐ and breed‐dependent, with no significant difference between the normal and diseased MCPs as identified on CT [27]. A study comparing MCP HU in dogs with arthroscopically confirmed MCPD and dogs with normal MCPs euthanized due to unrelated disease found that the MCP HU was significantly lower in the group with MCPD [28], and these results were comparable to two other studies [29, 30]. Another study assessing different diagnostic modalities for ED found that an increased or decreased HU and bone density could be associated with the presence of elbow pathology, and an increase at the MCP apex and base can be related to MCPD [6]. Sclerosis in the region of the MCP was most recently proposed to represent a breed or population variation and not necessarily ED. Therefore, MCP sclerosis without other evidence of ED should be interpreted with caution [31]. These studies each utilized a different method in determining MCP HU.

Limited studies have assessed radial head bone density in dogs with and without MCPD. Phillips et al. assessed radial bone density in LR (with and without MCPD) and ED‐free Greyhounds by measuring HU in each group [29]. Lower radial bone density was identified in the radial head adjacent to the MCP in the LR with a fragmented MCP compared to those without. They concluded that the changes seen in the radial head bone density suggested that it was also involved in the pathogenesis of MCPD.

Earlier identification of MCP abnormalities requires a complete understanding of its normal anatomy and appearance, and a better understanding could enable earlier identification of abnormalities to enable earlier intervention and improvement of the dog's welfare [20]. This retrospective descriptive study aimed to define the normal shape of the MCP on CT and determine if there was variability in the shape within the purpose‐bred LR. We further determined the normal range of HU of the MCP as well as that of the medial radial head (MRH) and lateral radial head (LRH). We hypothesized that there would be variation in the shape and HU of the normal MCP even in this uniform population of young adult LR.

## 2 Materials and Methods

### 2.1 Study Design and Population

This was a single‐center, retrospective descriptive study. Ethical approval was granted by the Research Ethics Committee of our institution (REC060‐23), and consent to use the collected data was obtained from the South African Guide Dog Association.

Imaging data collected between January 2019 and January 2023 were reviewed. Initial data included bilateral elbow radiographs and CTs of 278 guide dogs. The radiographs and CTs were previously assessed independently by European board‐certified radiologist (R.K.) with 30 years of experience. The following inclusion criteria resulted in a selection of 53 dogs or 106 elbows: LR, imaged at 12–13 months of either sex, with no history or signs of lameness at the time of imaging, did not have a surgical procedure to prevent ED and had no evidence of ED on either modality. Dogs were excluded if there was inadequate inclusion of the MCP on the CT images or if there were any equivocal changes on the MCP or radial head that may have indicated pathology.

### 2.2 CT Imaging Protocol

All dogs underwent helical CT scanning using a dual‐slice scanner (Siemens Emotion Duo with sliding gantry; Siemens Medical Systems, Forchheim, Germany) in dorsal recumbency with both legs scanned simultaneously. Dogs were sedated with medetomidine hydrochloride (Domitor, Orion Corporation, Orion Pharma, Finland) at 0.01 mg/kg intravenously and reversed with atipamezole hydrochloride (Antisedan, Orion Corporation, Zoetis, United States) at 0.003 mg/kg intramuscularly.

Technique settings included 1 mm thick transverse slices obtained in a bone algorithm (WW 1400, WL 300), kVp 130, effective mAs set at 70 with CAREDose selected, rotation time 1 s, and a pitch of 1. The initial acquisition was followed by individual elbow transverse reconstructions at 0.5 mm using an inner ear algorithm (WW 4000, WL 700).

Images were reviewed in digital imaging and communications in medicine (DICOM) image viewing software (Horos software, ver. 3.3.6, Horos Project, Minneapolis, MN, USA). Images were reconstructed using 3D multiplanar reconstruction (MPR). Images were not randomized, and reviewers were aware that they were all previously determined to be normal. Both elbows of each patient were evaluated by two reviewers, consisting of a second‐year diagnostic imaging resident (L.v.d.L., Reviewer 1) and a European board‐certified radiologist with 8 years of experience (C.L.R., Reviewer 2).

### 2.3 Elbow Alignment Technique

**FIGURE 1:**
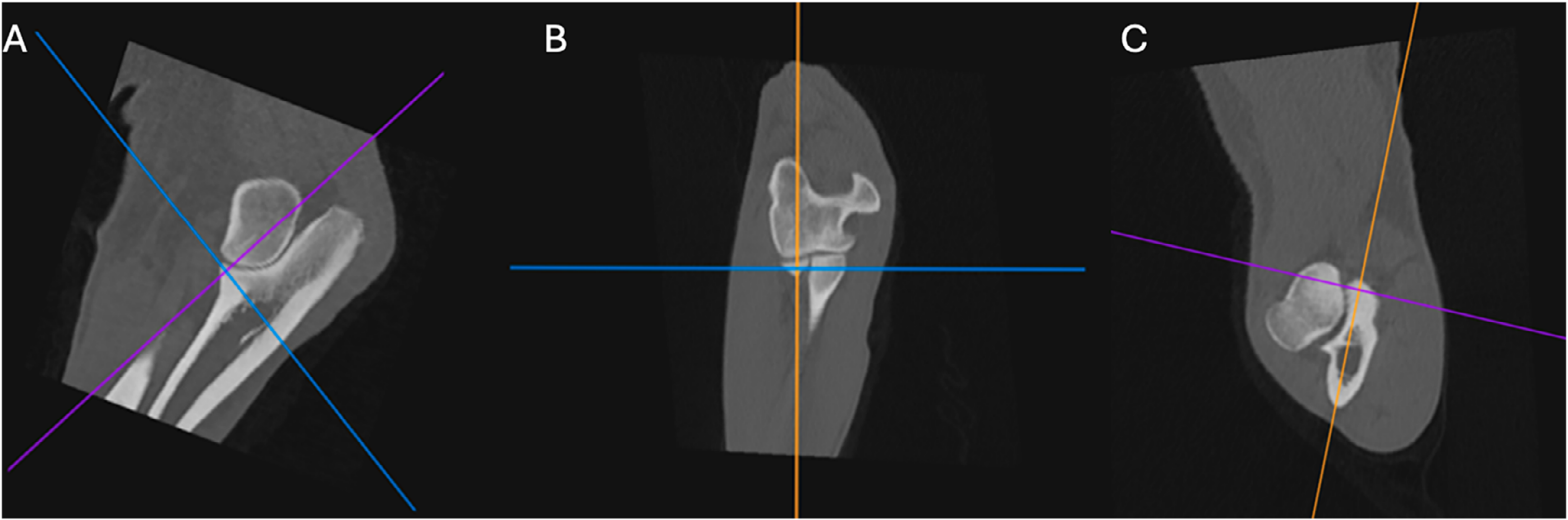
(A) Sagittal, (B) dorsal, and (C) transverse CT images of a 3D‐MPR demonstrating the alignment technique used in each elbow (WW 4000, WL 700).

**FIGURE 2:**
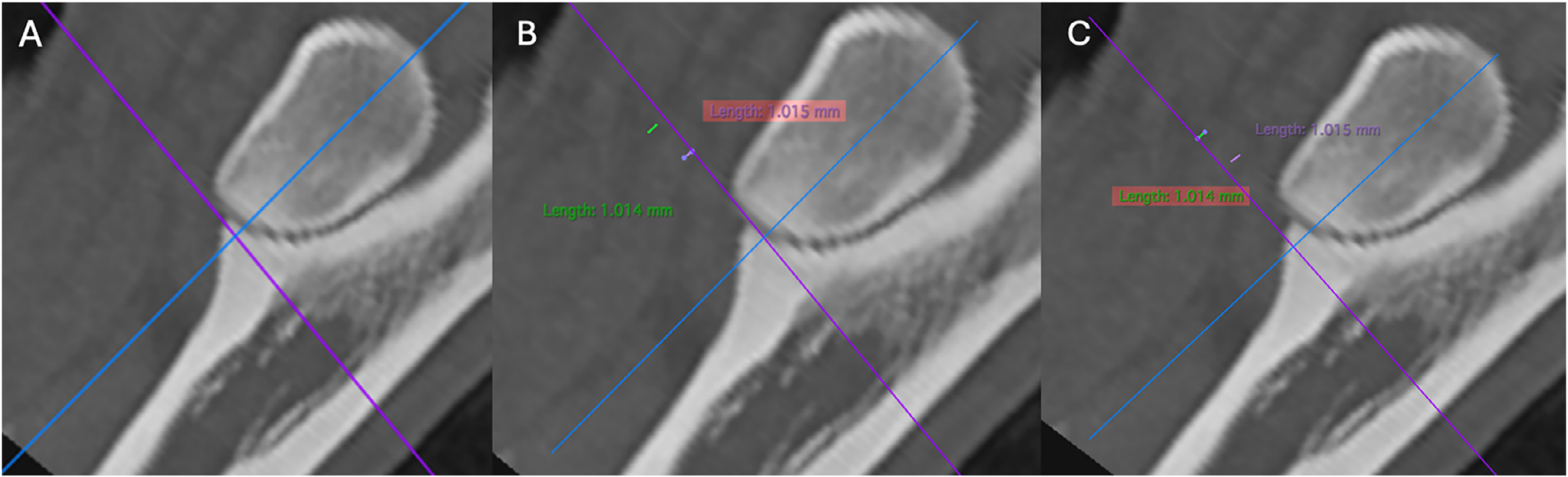
Examples of sagittal CT images of an elbow demonstrating the different levels that were evaluated: (A) MCP at level 0, (B) 1 mm was measured with the measuring tool above and below level 0 for each elbow to obtain level +1, and (C) level −1 (WW 4000, WL 700).

The elbow joints were aligned using a method adapted from two other studies, allowing the images to be assessed in a repeatable manner (Figure 1) [29, 32]. On the sagittal view, the dorsal slices (purple line) were aligned parallel with the caudal cortex of the ulna. The transverse slice (blue line) was lined up at the level of the tip of the apex of the MCP. On the dorsal view, the sagittal slice (orange line) was lined up parallel with the lateral aspect of the cortex of the ulna, and the midpoint of the transverse slice (blue line) and sagittal slice (orange line) was placed on the MCP. Lastly, on the transverse view, the midpoint of the sagittal (orange line) and dorsal slice (purple line) was placed at the apex of the MCP, and the sagittal slice (orange line) was rotated to cut halfway through the MCP and the caudal ulna cortex. This created a consistent image centered through the apex of the MCP and was identified as level 0. The MCP and radial head were then additionally evaluated at 1 mm above (+1) and 1 mm below (−1) level 0 by measuring this distance on the sagittal images and moving the transverse slice proximally or distally (Figure 2).

### 2.4 MCP Shape and HU Measurements of the MCP and Radial Head

**FIGURE 3:**
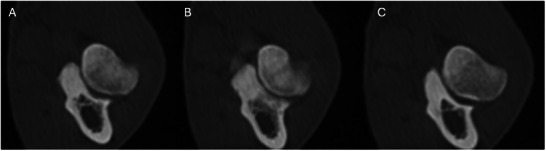
Transverse CT images of the medial coronoid process of the right elbow of the same female dog as examples of the three principal shape categories: (A) ovoid at level 0, (B) triangular at level +1, and (C) softly pointed at level −1 (WW 4000, WL 700).

After alignment, the outer margin of the ulnar cortex was traced at all three levels (0, −1, and +1), and images were saved as JPEGs. Once the images were collected, the shape of each MCP was classified into one of the three principal shape categories. This included ovoid, triangular, and softly pointed (Figure 3). The categories were established by consensus, based on the most frequently identified, easily understood, and most representative shapes. The medial margin and the cranial apex of the MCP were assessed to determine this shape. The “ovoid” shape was assigned when these were short and rounded, the “triangular” shape when the medial margin was flattened and the apex more pointed, and the “softly pointed” shape was assigned when the MCP had an ellipsoid apex and medial margin.

**FIGURE 4:**
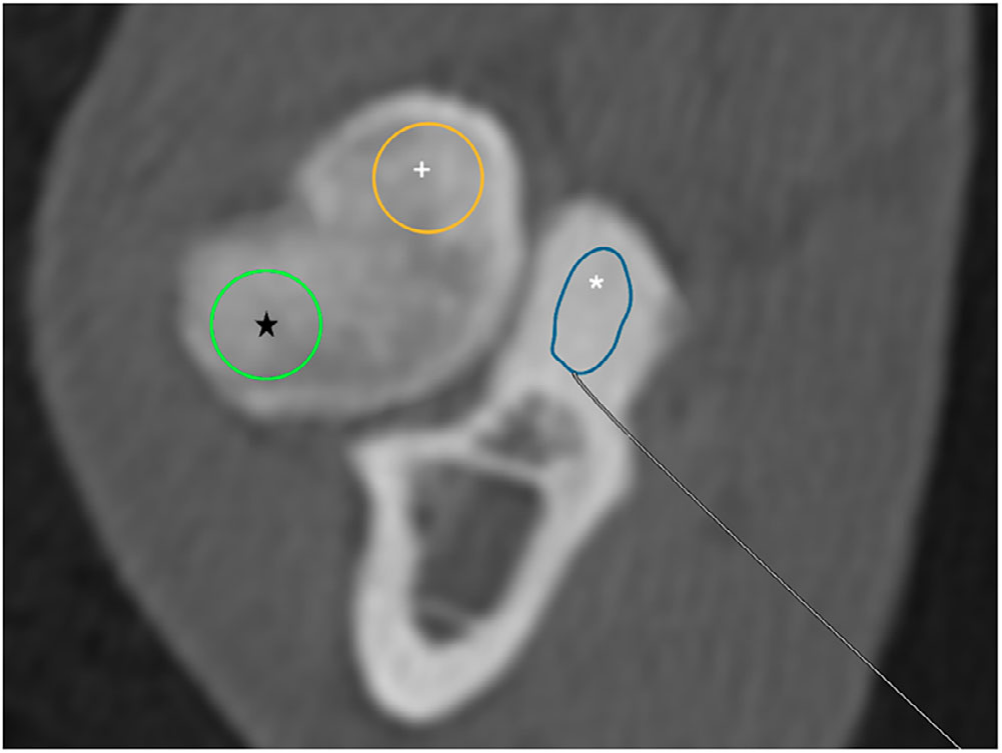
Transverse CT image of the medial coronoid process (MCP) and radial head depicting the different regions of interest (ROI) used to measure the HU of each. The blue ROI (asterisk) is a freehand tracing of the MCP just inside the ulna cortex using the pencil tool. The yellow ROI (plus) is a 5 mm diameter circular ROI inside the cortex on the medial radial head, and the green ROI (black star) measures the lateral radial head (WW 4000, WL 700).

The HU of each MCP at all three levels was measured using the freehand region of interest (ROI) pencil tool in the DICOM viewing software, drawing from the MCP apex to the base (excluding the ulnar medulla) just inside the cortex to avoid the effect the cortical bone could have on the HU (Figure 4). The radial head's comparative HU was measured at each level using a 5‐mm diameter circle‐shaped ROI tool, drawn just inside the cortex on both the MRH and LRH (Figure 4). At each level, the MCP, MRH, and LRH HU means were recorded in an Excel spreadsheet (Microsoft Excel, Microsoft Corp, Redmond, WA), and the values were compared. Both reviewers performed these measurements, and Reviewer 1 repeated the HU measurements 2 months later. Any technical concerns were additionally noted.

### 2.5 Statistics

Quantitative data were assessed for normality by calculating descriptive statistics, plotting histograms, and performing the Anderson–Darling test in commercial software (MINITAB Statistical Software, Release 13.32, Minitab Inc., State College, Pennsylvania, USA). Categorical data were described using frequencies, proportions (percentages), and 95% mid‐P exact confidence intervals (CIs). The shape of the MCP was compared between male and female dogs using Fisher's exact tests. Categorical data analysis was performed using available freeware (OpenEpi: Open Source Epidemiologic Statistics for Public Health. www.OpenEpi.com). Agreement between the two reviewers concerning the shape of the MCP was assessed using a Kappa statistic. Intra‐observer and interobserver repeatability for quantitative data were estimated using intra‐class correlation statistics. Quantitative measurements were compared over the three MCP levels using nonparametric Friedman tests. Mixed‐effects linear regression was used to estimate mean values and 95% CI, as well as identify significant predictors and compare quantitative measurements between male and female dogs. Each dog was included as a random effect with a variance components covariance structure in all models to account for the repeated measurements per study animal. Incorporated fixed effects were observer, MCP level, sex, and an interaction term between sex and MCP level. Unless otherwise stated, data analysis was implemented in commercially available software (IBM SPSS Statistics Version 29, International Business Machines Corp., Armonk, NY, USA) with a significance level of *p* < 0.05.

## 3 Results

### 3.1 Study Population

Of the 53 dogs that were enrolled in the study, 51 dogs, or 102 elbow joints, were included. Dogs were all purpose‐bred guide dog LRs with a mean age of 369 days (median 369, range 363–383 days) at the time of the CT study. All dogs were intact, of which 29 were females and 22 were males, from 31 litters, with one to two dogs from each litter.

### 3.2 MCP Shape

At level 0, both Reviewers 1 and 2 classified 101 (99.0%) MCPs as ovoid and one (1.0%) as triangular (Tables 1 and 2). At level +1, 101 (99.0%) MCPs were classified as triangular and one (1.0%) as ovoid by both reviewers. At level −1, Reviewer 1 classified 97 (95.0%) as softly pointed and 5 (5.0%) as ovoid, whereas Reviewer 2 classified 90 (88.2%) as softly pointed and 12 (11.8%) as ovoid.

**TABLE 1:** Descriptive presentation of the shape of the medial coronoid process (MCP) in 51, 1‐year‐old Labrador Retrievers from a single guide dog agency in South Africa.

			Male (*n* = 22)		Female (*n* = 29)		
Limb	Level	Shape	*n*	Percent (95% CI)	*n*	Percent (95% CI)	*p* value^a^
Left							
	−1	Ovoid	0	0 (0, 13)	3	10 (3, 26)	0.176
		Softly pointed	22	100 (87, 100)	26	90 (74, 97)	0.176
		Triangular	0	0 (0, 13)	0	0 (0, 10)	1.0
							
	0	Ovoid	22	100 (87, 100)	29	100 (90, 100)	1.0
		Softly pointed	0	0 (0, 13)	0	0 (0, 10)	1.0
		Triangular	0	0 (0, 13)	0	0 (0, 10)	1.0
							
	+1	Ovoid	1	5 (0, 20)	0	0 (0, 10)	0.431
		Softly pointed	0	0 (0, 13)	0	0 (0, 10)	1.0
		Triangular	21	95 (80, 100)	29	100 (90, 100)	0.431
							
Right							
	−1	Ovoid	0	0 (0, 13)	2	7 (1, 21)	0.318
		Softly pointed	22	100 (87, 100)	27	93 (79, 99)	0.318
		Triangular	0	0 (0, 13)	0	0 (0, 10)	1.0
							
	0	Ovoid	22	100 (87, 100)	28	97 (84, 100)	0.569
		Softly pointed	0	0 (0, 13)	0	0 (0, 10)	1.0
		Triangular	0	0 (0, 13)	1	3 (0, 16)	0.569
							
	+1	Ovoid	0	0 (0, 13)	0	0 (0, 10)	1.0
		Softly pointed	0	0 (0, 13)	0	0 (0, 10)	1.0
		Triangular	22	100 (87, 100)	29	100 (90, 100)	1.0

*Note*: Shape was determined by a second‐year radiology resident and compared between sexes.

Abbreviation: CI, confidence interval.

^a^ Based on Fisher's exact tests.

**TABLE 2:** Descriptive presentation of the shape of the medial coronoid process (MCP) in 51, 1‐year‐old Labrador Retrievers from a single guide dog agency in South Africa.

			Male (*n* = 22)		Female (*n* = 29)		
Limb	Level	Shape	*n*	Percent (95% CI)	*n*	Percent (95% CI)	*p* value^a^
Left							
	−1	Ovoid	2	9 (2, 27)	6	21 (9, 38)	0.294
		Softly pointed	20	91 (73, 98)	23	79 (62, 91)	0.297
		Triangular	0	0 (0, 13)	0	0 (0, 10)	1.0
							
	0	Ovoid	22	100 (87, 100)	29	100 (90, 100)	1.0
		Softly pointed	0	0 (0, 13)	0	0 (0, 10)	1.0
		Triangular	0	0 (0, 13)	0	0 (0, 10)	1.0
							
	+1	Ovoid	1	5 (0, 20)	0	0 (0, 10)	0.431
		Softly pointed	0	0 (0, 13)	0	0 (0, 10)	1.0
		Triangular	21	95 (80, 100)	29	100 (90, 100)	0.431
							
Right							
	−1	Ovoid	1	5 (0, 20)	3	10 (3, 26)	0.512
		Softly pointed	21	95 (80, 100)	26	90 (74, 97)	0.512
		Triangular	0	0 (0, 13)	0	0 (0, 10)	1.0
							
	0	Ovoid	22	100 (87, 100)	28	97 (84, 100)	0.567
		Softly pointed	0	0 (0, 13)	0	0 (0, 10)	1.0
		Triangular	0	0 (0, 13)	1	3 (0, 16)	0.567
							
	+1	Ovoid	0	0 (0, 13)	0	0 (0, 10)	1.0
		Softly pointed	0	0 (0, 13)	0	0 (0, 10)	1.0
		Triangular	22	100 (87, 100)	29	100 (90, 100)	1.0

*Note*: Shape was determined by a European board‐certified radiologist with 8 years of experience and compared between sexes.

Abbreviation: CI, confidence interval.

^a^ Based on Fisher's exact test.

At level 0 and +1, both reviewers agreed that the principal shapes between the left and right elbows were the same in 102 (100%) of the MCPs. At level −1, there were 7 (6.9%) disagreements between the reviewers, where Reviewer 1 identified the shape as softly pointed and Reviewer 2 as ovoid. The kappa (95% CI) for the agreement between reviewers concerning MCP shape was 0.97 (0.94, 0.99).

There was no statistically significant variation of the MCP shapes between the left and right elbows, at the different levels, or between females and males for either reviewer (Tables 1 and 2). Although not significantly different, the females were slightly more variable in shape than the males, and Reviewer 2 found more variations than Reviewer 1.

### 3.3 HUs of the MCP, MRH, and LRH

The HU of the MCP, MRH, and LRH varied over the three evaluated levels for both reviewers (*p* < 0.001) (Table 3).

**TABLE 3:** Descriptive presentation and comparison of radiological measures over three levels of the medial coronoid process (MCP) and radial head for 51, 1‐year‐old Labrador Retrievers from a single guide dog agency in South Africa.

			Level			
			−1	0	+1	
REV	Limb	Measure	Median (IQR)	Median (IQR)	Median (IQR)	*p* value^a^
REV1a	Left	MCP HU	1573 (1535, 1603)	1503 (1473, 1530)	1448 (1407, 1487)	<0.001^a^
		MRH HU	1165 (1073, 1221)	1220 (1133, 1289)	1283 (1201, 1351)	<0.001^a^
		LRH HU	675 (622, 711)	770 (663, 872)	876 (812, 936)	<0.001^a^
						
	Right	MCP HU	1580 (1549, 1614)	1523 (1481, 1550)	1480 (1433, 1509)	<0.001^a^
		MRH HU	1151 (1043, 1219)	1183 (1079, 1256)	1244 (1150, 1301)	<0.001^a^
		LRH HU	674 (615, 728)	749 (660, 836)	861 (780, 935)	<0.001^a^
						
REV1b	Left	MCP HU	1558 (1520, 1590)	1477 (1455, 1521)	1425 (1398, 1471)	<0.001^a^
		MRH HU	1172 (1090, 1263)	1211 (1111, 1279)	1278 (1197, 1338)	<0.001^a^
		LRH HU	681 (613, 718)	767 (662, 862)	867 (793, 952)	<0.001^a^
						
	Right	MCP HU	1571 (1536, 1592)	1498 (1460, 1530)	1442 (1411, 1487)	<0.001^a^
		MRH HU	1152 (1049, 1212)	1167 (1080, 1257)	1201 (1149, 1281)	<0.001^a^
		LRH HU	673 (616, 725)	786 (705, 895)	876 (794, 917)	<0.001^a^
						
REV2	Left	MCP HU	1535 (1486, 1560)	1474 (1446, 1503)	1418 (1387, 1448)	<0.001^a^
		MRH HU	1204 (1061, 1272)	1208 (1132, 1310)	1273 (1201, 1329)	<0.001^a^
		LRH HU	659 (622, 729)	790 (724, 890)	867 (779, 953)	<0.001^a^
						
	Right	MCP HU	1541 (1520, 1577)	1493 (1461, 1529)	1444 (1410, 1492)	<0.001^a^
		MRH HU	1188 (1103, 1244)	1213 (1132, 1285)	1286 (1224, 1334)	<0.001^a^
		LRH HU	679 (623, 741)	826 (749, 889)	843 (762, 928)	<0.001^a^

*Note*: REV1a, data collected by Reviewer 1 in Round 1; REV1b, data collected by Reviewer 1 in Round 2; REV2, data collected by Reviewer 2.

Abbreviations: HU, Hounsfield units; IQR, inter‐quartile range; LRH, lateral radial head; MRH, medial radial head; REV, reviewer.

^a^ *p* value based on Friedman tests.

The females were predicted to have a higher mean MCP HU compared to the males at all three levels (Table 4). The MCP HU was predicted to be highest in both sexes at level −1 and lowest at level +1. The range for the mean MCP HU for all three levels was 1501 to 1526 HU for the females and 1460 to 1490 HU for the males (95% CI) (Table 4). The mean HU of the MCP without sex segregation at level 0 was 1490 HU, at level +1 was 1438 HU, and at level −1 was 1556 HU.

**TABLE 4:** Model‐predicted radiological measures for three levels of the medial coronoid process (MCP) and radial head using mixed‐effects linear regression.

		Male (*n* = 22)		Female (*n* = 29)	
Level	Measurement	Mean^a^	95% CI^a^	Mean^a^	95% CI^a^
−1	MCP HU	1534	1518, 1550	1577	1563, 1591
	MRH HU	1137	1100, 1175	1170	1137, 1202
	LRH HU	648	619, 677	701	676, 727
					
0	MCP HU	1471	1455, 1487	1509	1495, 1523
	MRH HU	1176	1139, 1214	1211	1178, 1244
	LRH HU	733	703, 762	831	805, 856
					
+1	MCP HU	1420	1455, 1487	1455	1441, 1468
	MRH HU	1238	1201, 1276	1262	1230, 1295
	LRH HU	814	785, 844	893	868, 919
					
All	MCP HU	1475	1460, 1490	1513	1501, 1526
	MRH HU	1184	1148, 1219	1214	1183, 1245
	LRH HU	732	705, 759	808	785, 832

*Note*: Data were collected from 51, 1‐year‐old Labrador Retrievers from a single guide dog agency in South Africa.

Abbreviations: CI, confidence interval; HU, Hounsfield units; LRH, lateral radial head; MRH, medial radial head.

^a^ Based on mixed‐effects linear regression.

Similarly, females were predicted to have a higher mean MRH and LRH HU compared to the males at all three levels (Table 4). The mean MRH and LRH HU in both sexes were predicted to be highest at level +1 and lowest at level −1. The LRH consistently had a much lower predicted mean HU when compared to the MRH at all three levels for both sexes. The predicted mean HU of the MCP in both sexes was higher than the MRH and LRH HU at all three levels. The MRH HU for both reviewers across all three levels ranged from 1183 to 1245 HU for the females and 1148 to 1219 HU for the males (95% CI). The MRH HUs for the females were higher than that for the males, but the 95% CI overlapped. The LRH HU for both reviewers across all three levels ranged from 785 to 832 HU for the females and 705 to 759 HU for the males (95% CI). The LRH HUs for the females were higher than that of the males, with no observed overlap in the 95% CI.

The MCP HU varied by sex (*p* < 0.001), measurement level (*p* < 0.001), and observer (*p* < 0.001), but the effect of sex did not vary by measurement level (*p* = 0.418). The MRH HU did not vary by sex (*p* = 0.201), and the effect of sex also did not vary by measurement level (*p* = 0.727), but the HU did vary by measurement level (*p* < 0.001) and the observer (*p* < 0.001). The LRH HU varied by sex (*p* < 0.001), measurement level (*p* < 0.001), and the observer (*p* = 0.047), and the effect of sex varied by measurement level (*p* = 0.004).

The intra‐observer repeatability of the MCP HU at all three levels was good, and the interobserver repeatability was moderate at all three levels (Table 5). Level −1 had the best intra‐observer repeatability, and level +1 had the best interobserver repeatability. The intra‐observer repeatability for the MRH HU was excellent at level 0 and good at levels +1 and −1, and the interobserver repeatability was good at levels 0 and +1, and moderate at level −1. The intra‐observer repeatability for the LRH HU was good at level 0 and moderate at levels +1 and −1. The interobserver repeatability was moderate at all three levels; however, the repeatability of the LRH was not as good as that of the MRH.

**TABLE 5:** Intra‐observer and interobserver repeatability for two evaluators of radiological measures evaluated over three levels of the medial coronoid process (MCP) and radial head for 51, 1‐year‐old Labrador Retrievers from a single guide dog agency in South Africa.

		Level		
		−1	0	+1
Measurement	Repeatability	ICC (95% CI)	ICC (95% CI)	ICC (95% CI)
MCP HU	Intra‐observer	0.90 (0.85, 0.93)	0.81 (0.73, 0.86)	0.76 (0.67, 0.83)
	Interobserver	0.70 (0.59, 0.79)	0.63 (0.50, 0.73)	0.73 (0.62, 0.81)
				
MRH HU	Intra‐observer	0.87 (0.82, 0.91)	0.94 (0.92, 0.96)	0.89 (0.84, 0.92)
	Interobserver	0.61 (0.47, 0.72)	0.80 (0.72, 0.86)	0.78 (0.69, 0.85)
				
LRH HU	Intra‐observer	0.70 (0.59, 0.79)	0.82 (0.74, 0.87)	0.70 (0.59, 0.79)
	Interobserver	0.56 (0.41, 0.68)	0.54 (0.39, 0.67)	0.57 (0.42, 0.69)

Abbreviations: CI, confidence interval; HU, Hounsfield units; ICC, intra‐class correlation; LRH, lateral radial head; MRH, medial radial head.

### 3.4 Technical Factors

At level 0, Reviewer 1 identified six elbows (5.9%) with volume averaging, whereas Reviewer 2 only reported four (3.9%). At level +1, Reviewer 1 identified 66 (64.7%) and Reviewer 2 reported 73 (71.6%) with volume averaging. At level −1, Reviewer 1 reported six (5.9%), whereas Reviewer 2 only identified one (1.0%). Both reviewers identified volume averaging most frequently at level +1.

## 4 Discussion

The findings from this study demonstrate that the MCP has a particular shape that varies depending on where on the MCP it is assessed, when a standardized alignment technique is used. A standardized alignment technique was used to remove imaging orientation as an influencing factor when determining the MCP shape.

Three principal shapes were identified with excellent repeatability (kappa = 0.97) between the reviewers. The ovoid, triangular, and softly pointed shapes were, respectively, identified at level 0, +1, and −1 most frequently. Although there was no statistically significant variation in the shape between the elbows or sexes, the shape did vary depending on the level at which it was assessed. Most variation in shape was identified at level −1. Variations might have occurred due to a small margin of error during the placement of the guidelines. Level −1 was the last to be assessed therefore increasing the likelihood of slight deviations.

The study by Klumpp et al. identified the MCP as rounded, pointed, flattened, or irregular [27]. When we subjectively compared our shapes to theirs, it proved difficult to draw clear comparisons, although there were some similarities to our triangular shape. The CT MPR alignment technique in their study was standardized but differed from ours and was not specific for improved visualization of the MCP. On the basis of this, we concluded that the MCP shape can be influenced by the alignment technique.

Our study did not identify an irregular shape, likely due to our exclusion criteria. Specifically, dogs had to be ED‐free on both radiography and CT, whereas Klumpp et al. selected dogs only based on a radiological ED grade of 0 [27]. The sensitivity of radiography when diagnosing ED could have influenced their shapes, as dogs graded 0 on radiography can still be diagnosed with ED on CT [21, 24, 25].

On the basis of the authors’ experience, subjectively, the MRH has a slightly more hyperattenuating appearance than the LRH in elbows that are ED‐free, a finding supported by Phillips et al. [29]. On the basis of this study, zones corresponding to the LRH appeared to be the least affected by changes in bone density and had lower HUs than the MRH [29].

The mean MCP HU range for all three levels was 1501 to 1526 HU for females and 1460 to 1490 HU (95% CI) for males. Klumpp et al. divided their ED grade 0 dogs into two groups following elbow CT [27]. Group 1 had no evidence of MCP fissures or fragmentation, and Group 2 had fissures with or without fragmentation. They reported the mean HU of female LR to range from 1212 to 1554 HU in Group 1 and 1360–1485 HU in Group 2. The HU for the male dogs was not described in the previous article; the reason was unclear. As expected, our results for the females overlapped with Group 1, which was a more comparable population. The ulna cortex was included in their measurements, and a different MPR alignment technique was used, which could have influenced the HU measurement. We excluded the cortex in our measurements, as the medullary cavity is the region that subjectively most frequently demonstrates attenuation changes. The cortex presumably demonstrates fewer changes due to its inherent hyperattenuating appearance. Their inclusion criteria could also have played a role. The main difference is that their study included dogs based only on a radiographic ED grading of 0, without excluding CT abnormalities, whereas our population was additionally more stringently assessed for normality [27].

Our MCP HU, as for the shapes, varied across different levels, again demonstrating the significance of the level measured. In our study, the mean MCP HU, without sex segregation, at level 0 was 1490 HU, +1 was 1438 HU, and −1 was 1556 HU (95% CI), creating an increasing gradient from proximal to distal. A study by Humphreys et al. reported the mean MCP HU to be 1361.94 for cases with and without MCPD, 1606.56 HU for cases with no evidence of MCPD, and 1274.97 for cases with severe MCPD [28]. The results in their group with no evidence of MCPD were slightly higher than at our levels (50.56 HU higher than level −1). Their measurements included the ulna cortex, which could explain the higher HU. Their alignment technique was not described; various breeds and ages were included, and inter‐scanner variability should also be considered, which could have influenced their results [33]. Ellis et al. reported the mean MCP HU for LR cross GRs diagnosed ED‐free on CT to be 1537.45 HU, which was similar to our level −1 (1556 HU) [31]. A different alignment technique was used in their study, and the cortex was not included. This again demonstrated that the measurement level influenced the HU, even without including the cortex, but not accounting for scanner differences. The differences in the HU found by Humphreys et al. and Ellis et al. might also be attributed to the fact that not all dogs in these studies were LRs, with breed differences confirmed in the latter study, as well as the study by Klumpp et al. [27, 28, 31].

Although we found that HU varied at each level, they were still within a narrow range (females: 1501–1526 and males: 1460–1490 HU [95% CI]), and when compared to other studies, there was still some overlap between the normal and abnormal MCP HU [27, 28, 31]. We cannot confidently conclude that the overlap was due to scanner variables, differences in pre‐CT screening (for example, only including normal radiographs), ED severity, the alignment technique, or whether the cortex was included. It is, therefore, not possible to propose a normal HU range, and a comparison to LR with ED from the same population, excluding the cortex and using different MPR alignment techniques, would have to be evaluated.

The median MCP HU was found to be highest at level −1 and lowest at level +1 for both sexes. This could be due to higher bone density distally (−1), where it is narrower and where more compact bone can be appreciated compared to the more proximal levels (+1), where volume averaging may also have influenced the results. Females had a significantly higher mean MCP HU compared to males at all three levels. The study by Klumpp et al. demonstrated a significant sex dependence, but the possible reasons were not discussed [27]. Our study also demonstrated that the MCP HU can vary by sex in a population of ED‐free LR.

The HU of the MRH and LRH significantly varied over the three evaluated levels for both reviewers (*p* < 0.001). The MRH HUs for males were lower, but the 95% CI overlapped with that of the females. The median and mean HUs of the MRH were consistently higher than the LRH at all three levels for both sexes. Subjectively, the MRH was consistently more hyperattenuating relative to the LRH. The authors have also subjectively observed this on CT, regardless of whether the elbow was normal or affected. A CT osteoabsorptiometry study reported increased subchondral bone density in areas with increased load [34]. These areas included the craniomedial aspect of the radial joint surface, which correlates with our study findings, as well as other regions, such as the distomedial and cranial aspects of the humeral trochlea, within the olecranon fossa, and at the MCP and anconeal process of the ulna. According to Wolff's law, bone is a dynamic tissue that can remodel in response to mechanical load; therefore, increased bone density is seen in areas with increased load [35]. The higher MRH HUs were, therefore, attributed to the medial compartment bearing more weight than the lateral.

Across both reviewers, males had a lower mean MRH and LRH HU than females at all three levels. The lower HU values in males at the MCP, MRH, and LRH were unexpected, as a previous study reported that sex played a significant role in the weight distribution ratio between the thoracic and pelvic limbs, with females having a slightly lower mean than males across different breeds [36]. As per Wolff's law, it would be expected that the increased load in the males would cause increased bone density, but this was not observed. This might, therefore, be a normal variation within the breed [35]. For both reviewers, the mean MRH and LRH HU were highest in both females and males at level +1 and lowest at level −1. This could be because, at the subchondral level (closer to level +1) within the radial head, the bone density might be higher, because the load bearing will likely be higher the closer it is to the joint surface and cortex [35].

There was no overlap in LRH HU between the females and males, but this measurement was influenced by many variables. In the study performed by Phillips et al., radial bone density was assessed in ED‐free LR at six zones; zones corresponding to our MRH had the highest mean HU (1044.26 and 1130.75), and zones corresponding to our LRH had the lowest mean HU (762.25 and 739.61) [29]. The results are similar to our study and highlight the differences between the MRH and LRH.

Volume averaging was frequently encountered at level +1, which could have inadvertently influenced the HU, making this level less reliable. Therefore, the MCP HU is best evaluated at level −1, as it offers the best intra‐observer repeatability (good) with moderate interobserver repeatability and a small chance of volume averaging. When measuring radial head HU, the MRH at level 0 was best, given the MRH's lower variability, low risk of volume averaging, and good inter‐ and excellent intra‐observer repeatability.

The main limitations of this study were its retrospective nature and lack of arthroscopic or histological confirmation of the elbows as MCPD‐free. Arthroscopy is considered the gold standard technique in diagnosing MCPD but would have resulted in undesired and unacceptable morbidity in our population of non‐lame dogs found to be ED‐free on radiography and CT [17]. Similarly, histopathology would have been considered unethical. Another limitation is that dogs of various ages were not compared. It is generally accepted that the majority of physes in dogs will be closed by 12–13 months of age [37], and therefore, the MCP shape is not expected to change after this age in a dog without MCPD. However, bone density, including the MCP, does increase with age; therefore, the HU of the 1‐year‐old dog cannot be directly compared to other ages [6]. Our study did not include dogs diagnosed with MCPD, nor did it measure the MCP size. Breit et al. reported that the shape of the MCP varies between breeds; therefore, the results of our study should be used with caution when assessing different breeds [38]. Our study also did not include other commonly predisposed breeds. Due to our strict inclusion criteria, the sample size was small. CT‐associated limitations could also affect the HU measurements and should be considered, including the intra‐scanner variability and calibration technique used [33].

In conclusion, this study provides evidence that the MCP has a particular shape (ovoid, triangular, softly pointed), and the shape and HU are dependent on where on the MCP it is assessed and what alignment technique is used. The MCP HU varied by sex, with males having a significantly lower mean MCP HU compared to females. The mean HU of the MRH was consistently higher than the LRH, which could also be appreciated subjectively. The MCP HU is best evaluated at level −1, as it offers the best intra‐observer repeatability with moderate interobserver repeatability and a small possibility of volume averaging. The radial head HU is best evaluated on the MRH at level 0 as it has the lowest variability and risk of volume averaging, with good interobserver and excellent intra‐observer repeatability. This study also provides a reference for the MCP shape and HU in the young adult LR for future comparative studies.

## Author Contributions

**Robert M. Kirberger**: conception and design, acquisition of data, reviewing article for intellectual content, final approval of the completed article, agreement to be accountable for all aspects of the work in ensuring that questions related to the accuracy or integrity of any part of the work are appropriately investigated and resolved. **Christelle Le Roux**: conception and design, analysis and interpretation of data, reviewing article for intellectual content, final approval of the completed article, agreement to be accountable for all aspects of the work in ensuring that questions related to the accuracy or integrity of any part of the work are appropriately investigated and resolved. **Luzanne van der Laan**: analysis and interpretation of data, drafting the article, reviewing article for intellectual content, final approval of the completed article, agreement to be accountable for all aspects of the work in ensuring that questions related to the accuracy or integrity of any part of the work are appropriately investigated and resolved. **Geoffrey T. Fosgate**: analysis and interpretation of data, reviewing article for intellectual content, final approval of the completed article, agreement to be accountable for all aspects of the work in ensuring that questions related to the accuracy or integrity of any part of the work are appropriately investigated and resolved.

## Disclosure

The authors have nothing to report.

## Conflicts of Interest

The authors declare no conflicts of interest.

## Data Availability

The data from this study are available from the corresponding author upon reasonable request.
